# Inflammation and endothelial function relevant genetic polymorphisms in carotid stenosis in southwestern China

**DOI:** 10.3389/fneur.2022.1076898

**Published:** 2023-01-04

**Authors:** Lin Liu, Xingyang Yi, Hua Luo, Ming Yu

**Affiliations:** ^1^Department of Neurology, The People's Hospital of Deyang City, Deyang, China; ^2^Department of Neurology, The Affiliated Hospital of Southwest Medical University, Luzhou, China; ^3^Department of Neurology, The Suining Central Hospital, Suining, China

**Keywords:** stroke, high-risk population, inflammation, carotid stenosis, genetic polymorphism

## Abstract

**Aim:**

To evaluate the relationship between carotid stenosis with variants in genes referred to inflammation and endothelial function.

**Methods:**

There was a multi-center, cross sectional survey in southwestern China. The eight communities were selected at random in southwestern China. The residents aged ≥40 years volunteered to participate in face-to-face survey. Subjects with at least three of the aforementioned eight stroke related risk factors or a history of stroke were classified as high-risk population for stroke. A total of 2,377 subjects were the high-risk population for stroke in the eight communities, and degree of carotid stenosis was assessed by carotid ultrasound. Genotypes of 6 variants in 3 genes related to inflammation and endothelial function were examined. Gene-gene interaction was analyzed by generalized multifactor dimensionality reduction (GMDR).

**Results:**

Carotid stenosis were found in 295 (12.41%) subjects, of whom 51 (17.29%) had moderate or severe stenosis. According to multivariate logistic regression analysis, we found that HABP2rs7923349TT was independent risk factor for carotid stenosis (OR, 1.96, 95% CI: 1.22–3.13, *P* = 0.005) and ITGA2rs1991013AG and HABP2rs7923349TT were independent risk factors for moderate to severe carotid stenosis (OR, 2.28, 95% CI: 1.28–4.07, *P* = 0.005; OR, 2.90, 95% CI: 1.19–7.08, *P* = 0.019). GMDR analysis showed that there was a significant gene-gene interaction between *ITGA2* rs4865756 and *HABP2* rs7923349, and the high-risk interactive genotype in the two variants was independently associated with a higher risk for carotid stenosis after adjusting the covariates (OR,1. 42, 95% CI 1.10–1.84, *P* = 0.008).

**Conclusions:**

Prevalence of carotid stenosis was very high in the high-risk stroke population in southwestern China. Variants in genes referred in endothelial function were associated with the carotid stenosis. The high—risk interactive genotype in *ITGA2* rs4865756 and *HABP2* rs7923349 was independently associated with a higher risk for carotid stenosis.

## Introduction

Stroke is one of the leading causes of disability and mortality in China (1). A report has proven that stroke recurrence or cardiovascular outcomes are influenced by serious carotid stenosis, and it has been well-established in the population with stroke (2, 3). Moreover, severe internal carotid artery stenosis (≥50%) is usually accompanied by carotid atherosclerosis and emboli, thereby reducing blood flow of the brain, which increases the risk of stroke as well (4, 5). It is critical to clarify the etiology of carotid stenosis, particularly in the genetic etiology. Further understanding of the roles of genetic factors in carotid stenosis can provide better insight into the pathogenesis of this disease, to better preventing stroke occurrence.

Atherosclerosis is a process of chronic inflammation (6, 7). Carotid plaque, a phenotype of subclinical atherosclerosis, is an inflammatory lesion associated with stroke (8). Variants in inflammation and endothelial function genes have been reported to play roles in carotid plaques in the Dominican population (8). In addition, several studies have revealed that genes implicated in inflammation and endothelial function are associated with the occurrence, stability and vulnerability of carotid plaques (9–11). However, few studies have focused on genetic mechanism of carotid stenosis. According to the China National Stroke Screening Survey (CNSSS), a total of 2,377 subjects were the high-risk population for stroke in the eight communities in this study. Extracranial carotid artery stenosis was assessed by carotid ultrasound. In following article, extracranial carotid artery stenosis was abbreviated as carotid stenosis. Meanwhile, genotypes of 6 variants in 3 genes related to inflammation and endothelial function were examined. The aim of the present study was to evaluate the morbidity of carotid stenosis in a population with high stroke risk, the association between the 6 variants and carotid stenosis, and the effect of gene-gene interactions among the 6 variants on carotid stenosis according to this community-based study.

## Materials and methods

### Study population

This study was part of the CNSSS (Grant No. 2011BAI08B01) which was approved by the Stroke Screening and Prevention Programme of the National Health and Family Planning Commission of China. The survey protocol was approved by the ethics committee of the participating hospitals (The People's Hospital of Deyang City, the Affiliated Hospital of Southwest Medical University and Suining Central Hospital), Written informed consent was obtained from each subject prior to study enrollment.

During May 2015 and September 2015, eight communities in Sichuan were randomly selected. More details on the organization and implementation can be found on the official websites (12) and the literature we published before (13). We only screened all residents aged ≥40 years who lived in the community for more than 6 months. A structured face-to-face questionnaire was used in the initial screening by interviewers.

### Evaluation of risk factors and high stroke risk

Information on all the participants was collected, including demographic characteristics (e.g., age, sex), stroke-related behavioral factors (e.g., smoking, exercise habits and diet), personal and family medical history of stroke and chronic diseases (e.g., hypertension, diabetes mellitus, dyslipidemia and atrial fibrillation [AF]), and physical examination (e.g., height, weight).

The eight conventional stroke risk factors were assessed in the CNSSS questionnaire, including hypertension, diabetes, dyslipidemia, AF, overweight/obesity, smoking, physical inactivity, and family history of stroke. The detailed information of the participants was described in our previous article (13). Subjects were classified as the high-risk population for stroke if they experienced at least three of the aforementioned eight stroke related risk factors or a history of stroke. Exclusion criteria included: (i) severe cardiovascular, liver or renal disease, (ii) blood system disease and coagulation dysfunction, (iii) acute and chronic inflammation, malignant tumors and immune system diseases, (iv) transient ischemic attack only, and (v) individuals declined to participate in the study.

### Data cleaning procedures and quality control

**Figure 1:**
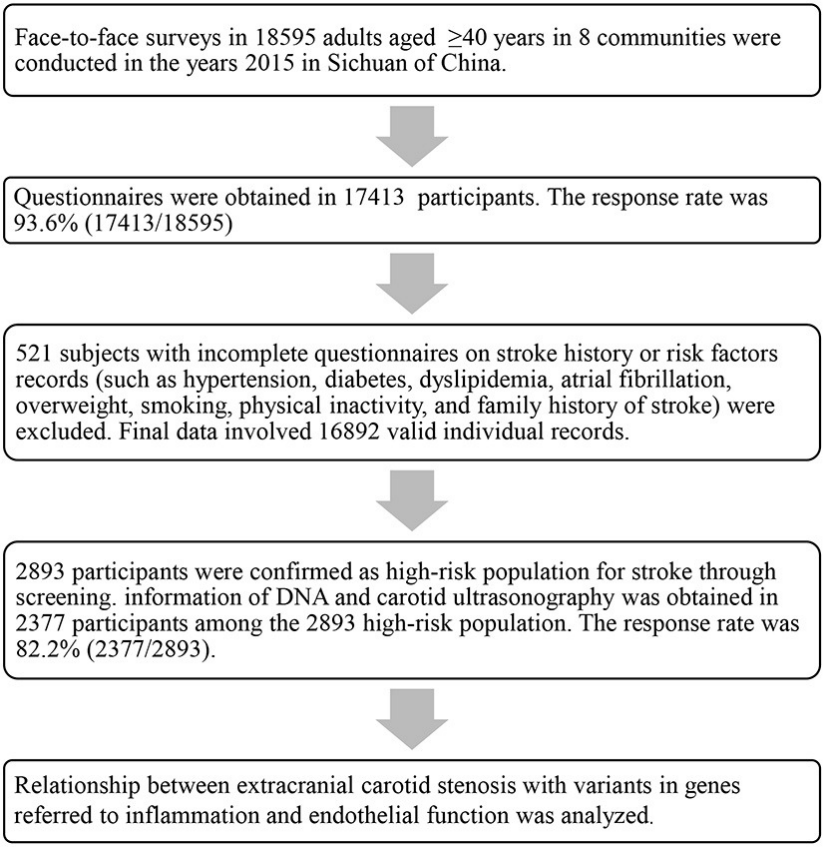
Research method and data cleaning flow chart in this study.

Finally, 16,892 valid individual records (including 524 stroke cases [429 ischemic strokes, 95 hemorrhagic strokes]) were enrolled. Among them, 2,893 participants were the high-risk population. However, DNA and carotid ultrasonography information was obtained in 2,377 participants among the 2,893 high-risk population. The detailed data cleaning procedure and quality control is presented in Figure 1.

### Carotid ultrasonography

Color duplex scans (Acuson Sequoia Apparatus type 512, 7.5-MHz probe, Berlin, Germany) were performed to evaluate the severity of extracranial carotid artery stenosis. Then we acquired longitudinal and transverse sections from color duplex imaging and power mode. Carotid stenosis assessment in diameter was combined with peak systolic velocities at the location of the stenosis as well as the internal carotid artery/common carotid artery ratio (14).

A total of 2,377 subjects were classified into carotid stenosis and non-carotid stenosis (< 15% stenosis) groups on the basis of their vascular imaging. According to degree of carotid stenosis, the subjects were further divided into moderate to severe stenosis group (≥50% stenosis) and non-moderate to severe stenosis group (< 50% stenosis) that includes mild stenosis group (15–49% stenosis) and non-carotid stenosis (< 15% stenosis) groups. Carotid ultrasound was conducted by sonographers blinded to the laboratory and questionnaire data.

### Genotyping

Six SNPs involved in inflammation and endothelial function were selected (https://www.ncbi.nlm.nih.gov/snp) according to the following criteria: (i) minor allele frequency>0.05; (ii) nonsynonymous variants; (iii) SNPs that have been assessed in previous studies (8). Whole blood (3 ml) collection, genomic DNA extraction, and genotyping were performed as previously described (10, 11, 13). Each SNP was designed with two amplification primers and one extension primer. According to our previous study, genotyping of the 6 variants was performed using the matrix-assisted laser desorption/ionization time of flight mass spectrometry (MALDI-TOF MS) method (10, 11, 13).

### Statistical analysis

All statistical analyses were performed using SPSS 16.0 (SPSS Inc, Chicago, IL, USA). Deviation of Hardy–Weinberg equilibrium for genotype frequencies was analyzed by χ^2^ test. Continuous variables conforming to a normal distribution are expressed as the mean ± standard deviation, and intergroup differences were evaluated using Student's *t*-test or analysis of variance. Categorical variables that were not in accord with a normal distribution were expressed as median and Interquartile range, and the rank sum test was used for comparison between groups. Categorical variables are presented as frequencies or percentages; intergroup differences were evaluated using the χ^2^ test or Fisher's exact test. Multivariate logistic regression analysis was performed to adjust covariate variables (age, gender, hypertension, diabetes, smoking, and hyperlipidemia) and to assess the independent risk factors for carotid stenosis, and odds ratios (ORs) with 95% confidence intervals (CIs) were calculated. All tests were 2 sided, and the threshold level of *P* < 0.05 denoted statistical significance.

Gene-gene interactions were analyzed using the statistical software GMDR Beta, version 0.7 (15), as described in our previous studies (11, 16). The GMDR computed the maximum likelihood estimates and the scores of all individuals under the null hypothesis. A cumulative score was then calculated within each multifactor cell, which was labeled either as high-risk if the average score met or exceeded a pre-assigned threshold of zero, or as low-risk if the score was less than zero. An exhaustive search of all possible one- to six-locus models was performed for all variants. The model with the minimum prediction error, the maximum cross-validation consistency score, and a *P*-value of 0.05 or less (derived automatically from the sign test in the GMDR software) was considered as the best model.

## Results

### Baseline characteristics and morbidity of carotid stenosis

Among the 2,377 high-risk population, 295 cases (12.41%) had carotid stenosis, of which 51 (17.29%) subjects had moderate to severe stenosis.

Compared with subjects without carotid stenosis, subjects with carotid stenosis were older and had a higher proportion of men, a history of stroke, cigarette smoking and taking antihypertensive drugs (Table 1). Subjects with moderate to severe carotid stenosis had a higher proportion of men and a history of stroke than subjects without moderate to severe carotid stenosis (Table 1).

**Table 1:** Demographic characteristics of the study population.

**Characteristics**	**Non-carotid stenosis (*n* = 2,082)**	**Carotid stenosis (*n* = 295)**	***P_1_*-value**	Non-moderate to severe carotid stenosis^*^(*n* = 2,326)	**Moderate to severe carotid stenosis (*n* = 51)**	***P_2_*-value**
Age (years)	62.93 (56.00–70.00)	66.52 (60.00–73.00)	0.000	64.00 (57.00–70.00)	65.00 (60.00–71.00)	0.226
Men (*n*, %)	906 (43.52)	159 (53.90)	0.001	1,035 (44.50)	30 (58.82)	0.042
Body mass index (kg/m^2^)	26.19 (23.53–28.44)	26.59 (23.34–28.06)	0.171	26.35 (23.50–28.43)	25.33 (23.37–27.59)	0.157
Atrial fibrillation (*n*, %)	46 (2.20%)	4 (1.36%)	0.339	49 (2.11)	1 (1.96)	1.000
History of stroke (*n*, %)	369 (17.72)	75 (25.42)	0.001	419 (18.01)	25 (49.02)	0.000
Hypertension (*n*, %)	1,581 (75.94)	222 (75.25)	0.798	1,769 (76.05)	34 (66.67)	0.121
Diabetes mellitus (*n*, %)	576 (27.67)	77 (26.10)	0.573	644 (27.69)	9 (17.65)	0.112
Rates of dyslipidemia (*n*, %)	692 (33.24)	83 (28.14)	0.080	757 (32.55)	18 (35.29)	0.679
Cigarette smoking (*n*, %)	689 (33.09)	127 (43.05)	0.001	797 (34.26)	19 (37.25)	0.656
**In-hospital treatment (** ***n*** **, %)**						
Antihypertensive drugs	813 (39.05)	137 (46.44)	0.015	934 (40.15)	16 (31.37)	0.205
Hypoglycemic drugs	365 (17.53)	46 (15.59)	0.410	407 (17.50)	4 (7.84)	0.071
Statins	160 (7.68)	14 (4.75)	0.070	174 (7.48)	0 (0.00)	0.079
Antiplatelet drugs	369 (17.72)	42 (14.24)	0.138	402 (17.28)	9 (17.65)	0.946

^*^Non-moderate to severe carotid stenosis group includes non-carotid stenosis and mild carotid stenosis groups.

### Genotype distributions in the groups with or without carotid stenosis

The genotype distributions of these 6 variants were in Hardy–Weinberg Equilibrium (all *P* > 0.05). Univariate analyses revealed that *HABP*2 rs7923349 was associated with carotid stenosis (*P* < 0.05, Table 2). The genotypes of *ITGA2* rs1991013 and *HABP2* rs7923349 were associated with moderate to severe carotid stenosis (*P* < 0.05, Table 3).

**Table 2:** Genotype distribution in individuals with and without carotid stenosis (*n* [%]).

**Genotype**	**Non-carotid stenosis (*n* = 2,082)**	**Carotid stenosis (*n* = 295)**	***p*-value**
IL1Ars1609682			0.134
GG	906 (43.52)	127 (43.05)	
GT	1,031 (49.52)	138 (46.78)	
TT	145 (6.96)	30 (10.17)	
IL1Ars1800587			0.123
AA	15 (0.72)	0 (0)	
AG	267 (12.82)	47 (15.93)	
GG	1,800 (86.46)	248 (84.07)	
ITGA2rs1991013			0.701
AA	966 (46.40)	142 (48.14)	
AG	910 (43.71)	128 (43.39)	
GG	206 (9.89)	25 (8.47)	
ITGA2rs4865756			0.069
AA	131 (6.29)	25 (8.47)	
AG	767 (36.84)	122 (41.36)	
GG	1,184 (56.87)	148 (50.17)	
HABP2rs7923349			0.000
GG	1,224 (58.79)	148 (50.17)	
GT	769 (36.94)	119 (40.34)	
TT	89 (4.27)	28 (9.49)	
HABP2rs932650			0.874
CC	200 (9.61)	31 (10.51)	
CT	902 (43.32)	128 (43.39)	
TT	980 (47.07)	136 (46.10)	

**Table 3:** Genotype distribution in moderate to severe carotid stenosis group and the group without moderate to severe carotid stenosis (*n* [%]).

**Genotype**	**Non-moderate to severe carotid stenosis (*n* = 2,326)**	**Moderate to severe carotid stenosis (*n* = 51)**	***p*-value**
IL1Ars1609682			0.536
GG	1,012 (43.51)	21 (41.18)	
GT	1,141 (49.05)	28 (54.90)	
TT	173 (7.44)	2 (3.92)	
IL1Ars1800587			0.844
AA	15 (0.64)	0 (0.00)	
AG	307 (13.20)	7 (13.73)	
GG	2,004 (86.16)	44 (86.27)	
ITGA2rs1991013			0.017
AA	1,091 (46.90)	17 (33.33)	
AG	1,006 (43.25)	32 (62.75)	
GG	229 (9.85)	2 (3.92)	
ITGA2rs4865756			0.273
AA	155 (6.66)	1 (1.96)	
AG	866 (37.23)	23 (45.10)	
GG	1,305 (56.10)	27 (52.94)	
HABP2rs7923349			0.032
GG	1,349 (58.00)	23 (45.10)	
GT	866 (37.23)	22 (43.14)	
TT	111 (4.77)	6 (11.76)	
HABP2rs932650			0.477
CC	226 (9.72)	5 (9.80)	
CT	1,012 (43.51)	18 (35.29)	
TT	1,088 (46.78)	28 (54.90)	

### Gene-gene interaction and its association with carotid stenosis

The associations of gene-gene interactions among the 6 variants with carotid stenosis were performed by the GMDR approach (Table 4). The best model for carotid stenosis including *ITGA2* rs4865756 and *HABP2* rs7923349 scored 8/10 for cross-validation consistency and 10/10 for sign testing. The *P*-value for prediction error was 0.010 for GMDR using permutation testing.

**Table 4:** Comparison of the best models, prediction accuracies, cross-validation consistencies, and *P*-values identified by GMDR.

Best model^*^	**Training balanced accuracy**	**Testing balanced accuracy**	**Sign test (*P*)**	**Cross-validation consistency**
1	0.5436	0.5289	7 (0.1719)	9/10
12	0.5579	0.5286	10 (0.0010)	8/10
123	0.5870	0.5346	10 (0.0010)	5/10
1,345	0.6174	0.5290	8 (0.0547)	7/10
12,345	0.6620	0.5301	8 (0.0547)	10/10
1,23,456	0.6777	0.5237	6 (0.3770)	10/10

^*^rs7923349, rs4865756, rs1609682, rs1991013, rs932650 and rs1800587 are symbolized as 1-6, respectively.

Cross-validation consistency ≤ 5/10 is not statistically significant.

GMDR, generalized multifactor dimensionality reduction.

### Different genotype combinations and the risk of carotid stenosis

Subsequently, we assessed the associations of different genotype combinations in *ITGA*2 rs4865756 and *HABP*2 rs7923349 with the risk of carotid stenosis.

Compared to patients harboring wild type genotypes (rs4865756GG, rs7923349GG), the relative risk of different genotype combinations of rs4865756 and rs7923349 was analyzed. The 5 genotype combinations making larger contributions to carotid stenosis risk were ITGA2rs4865756AA, HABP2rs7923349GG; ITGA2rs4865756AG, HABP2rs7923349GT; ITGA2rs48657 56AG, HABP2rs7923349TT; ITGA2rs4865756GG, HABP2rs7 923349GT and ITGA2rs4865756GG, HABP2rs7923349TT, which were considered as the high-risk interactive genotypes. The other combination genotypes among rs4865756 and rs7923349 did not reach statistical significance (*P* > 0.05) and were defined as the low-risk interactive genotype.

### Risk factors of carotid stenosis

Multivariate logistic regression analysis was performed to assess the risk of carotid stenosis conferred by different genotype combinations among rs4865756 and rs7923349. The incidence of carotid stenosis was significantly higher in subjects with the high-risk interactive genotype than in those carrying the low-risk interactive genotype [15.14 (160/1,057) vs. 10.23% (135/1,320), χ^2^ = 13.017, *P* < 0.001]. The high-risk interactions were assigned as one, and the low-risk interactions were assigned as zero. The other variables that showed a significant association with carotid stenosis (*P* < 0.05) on univariate analysis were enrolled in the multivariate logistic regression model. The results revealed that the high-risk interactions were independently associated with a higher risk for carotid stenosis after adjustment with covariates (OR, 1.42, 95% CI: 1.10–1.84, *P* = 0.008, Table 5). Besides, *HABP*2rs7923349TT was considered be an independent risk factor for carotid stenosis (OR, 1.96, 95% CI: 1.22–3.13, *P* = 0.005, Table 5).

**Table 5:** Multivariate analysis of the major risk factors for carotid stenosis.

**Risk factor**	***p-*value**	**OR**	**95%CI**
Age	0.000	1.04	1.02–1.05
Men	0.013	1.48	1.09–2.02
History of stroke	0.003	1.56	1.17–2.10
Smoke	0.123	1.28	0.94–1.76
Antihypertensive drugs	0.031	1.33	1.03–1.71
*HABP*2rs7923349TT	0.005	1.95	1.22–3.13
High-risk interactions	0.008	1.42	1.10–1.84

However, the incidence of moderate to severe carotid stenosis had no statistical differences between subjects with the high-risk interactive genotype and the low-risk interactive genotype [2.55 (27/1,057) vs. 1.82% (24/1,320), χ^2^ = 1.515, *P* = 0.218]. Multivariate logistic regression analysis was also performed to determine the risk factors for moderate to severe carotid stenosis using variables with *P*-values < 0.05 in univariate analysis, and found that *ITGA2*rs1991013AG and *HABP*2rs7923349TT were independent risk factors for moderate to severe carotid stenosis (OR, 2.28, 95% CI: 1.28–4.07, *P* = 0.005; OR, 2.90, 95% CI: 1.19–7.08, *P* = 0.019, Table 6).

**Table 6:** Multivariate analysis of the major risk factors for moderate to severe carotid stenosis.

**Risk factor**	***p-*value**	**OR**	**95%CI**
Men	0.007	2.20	1.24–3.92
History of stroke	0.000	4.99	2.82–8.84
*ITGA2*rs1991013AG	0.005	2.28	1.28–4.07
*HABP*2rs7923349TT	0.019	2.90	1.19–7.08

## Discussion

In this study, the relationships among baseline characteristics, genic interactions and carotid stenosis in high-risk stroke population were investigated. We detected a high prevalence (12.41%) of carotid stenosis among the high-risk population for stroke in southwestern China. By multivariate analysis, we found that one endothelium-associated gene loci (*HABP2* rs7923349TT) was associated with occurrence of carotid stenosis and two endothelium-associated gene loci (*ITGA2* rs1991013AG, *HABP2* rs7923349TT) were associated with occurrence of moderate to severe carotid stenosis.

Atherothrombosis is a main pathomechanism in the evolution of vessel stenosis (17). In recent years, increasing evidence suggests that atherosclerosis is an active process akin to the chronic inflammatory process adjusted by multiple genes (6, 7).

Integrin α2 (integrin alpha2), encoded by ITGA2, is a significant member of the integrin family and is connected with platelet adhesion and aggregation through collagen receptors. The ITGA2 gene polymorphism may be a susceptible predictor of the risk of ischemic stroke (18). However, Georgios K. Nikolopoulos et al. hold a different opinion. In their meta-analysis, there was no evidence to support an association between the C807T polymorphism of the ITGA2 gene and stroke (19). But a study about Dominicans found that *ITGA2* rs1991013 is associated with calcified plaque and increases the risk of general atherosclerosis (8). This conclusion is in accord with our result. In our research, *ITGA2* rs1991013 is associated with moderate to severe carotid stenosis. But number of moderate to severe carotid stenosis is limited, so further study is need.

HABP2, which encodes an extracellular serine protease involved in coagulation, fibrinolysis and inflammatory pathways, may be a genetic susceptibility locus in early-onset stroke (20). Meanwhile, HABP2 is believed to affect vascular smooth muscle cell proliferation and atherosclerotic plaque instability (21). This study detects that *HABP2* rs7923349 SNPs are related to carotid stenosis and moderate to severe carotid stenosis in people who are in high risk for stroke. The reason may be that HABP2 polymorphism is associated with inhibited activation of pro-urokinase and progression of carotid stenosis.

Many studies have explained the relationship between ischemic stroke and genes involved in inflammation and the endothelium. Yi et al. (22) discovered that eleven variants of platelet activation-relevant genes are unrelated to carotid stenosis, but gene-gene interactions among *TXA2R* rs1131882, *P2Y1* rs1371097 and *GPIIIa* rs2317676 have a synergistic influence on carotid stenosis by GMDR analysis. This indicates that a single-locus analytical approach seemed unfit for the genetic etiology of carotid stenosis. Atherosclerosis may be caused by gene-gene or gene-environment interactions (23, 24). Therefore, the genetic risk of carotid stenosis can be investigated gene-gene interactions *via* the GMDR approach (15). This study focuses on subclinical carotid stenosis which has received less attention before. According to GMDR analysis, we detected gene-gene interactions at the genetic locus between *ITGA2* rs4865756 and *HABP2* rs7923349. High-risk interaction genotypes increase the risk of carotid stenosis. However, the specific molecular mechanisms of gene-gene interactions among the 2 variants are still unclear. The reason may be that the two genes are involved in encoding and adjusting enzymes that cause atherosclerosis and refer to endothelial function, thereby resulting in the occurrence and development of arteriostenosis. The function of adhesion and aggregation could be increased by gene polymorphisms in *HABP2* rs7923349 and *ITGA2* rs4865756. Moreover, vascular integrity and atherosclerotic plaques are regulated by these genes (8). The relationship between genes related to endothelial and inflammatory function and carotid stenosis was investigated in China for the first time. The study discovered gene-gene interactions among *HABP2* rs7923349 and *ITGA2* rs4865756, and high-risk interaction genotypes increase the risk of carotid stenosis. New targets for preventing and treating carotid stenosis may be found in this research. However, the connection between high-risk interaction genotypes and moderate to severe carotid stenosis haven't been found in univariate analysis or multivariate analysis. The deficiency of number in participants or follow-up studies may be the reason. Therefore, it is meaningful to explore the theory of this research more deeply.

As we know, IL-1α from macrophage is a principal cytokine controlling the development of atherosclerotic plaques (25). But, in our study, genotypes of IL-1α were irrelevant to carotid stenosis. Besides, we found that antihypertensive therapy is positively associated with carotid stenosis in multivariate analysis. It's different from our inertial thinking and experience. The factors that contribute to this situation include that subjects with carotid stenosis have a higher proportion of hypertension than another group (Table 1) and people with carotid stenosis may have better compliance in taking antihypertensive. But further research and follow-up visit are needed.

Several potential limitations need to be considered in our findings. First, our study used a cross-sectional study design and self-report questionnaire; therefore, recalling bias might play roles in affecting the validity of our results. Second, carotid stenosis was assessed by ultrasound in our study. Although ultrasound can recognize carotid stenosis, Magnetic Resonance Angiography (MRA), computed tomography angiography (CTA) and digital subtraction angiography (DSA) may provide more detail about carotid stenosis. Third, we acknowledge that although the current study examined the roles of several important genes related to endothelial and inflammatory responses, other relevant genes were not examined. Fourth, in this study, even though gene-gene interactions between *HABP2* rs7923349 and *ITGA2* rs4865756 increased the risk of carotid stenosis, the detailed mechanism was unknown. Additionally, further studies are needed to clarify their role in carotid stenosis formation.

## Conclusion

In conclusion, the prevalence of carotid stenosis was high among the high-risk stroke population in southwestern China. We identified variants in genes related to endothelial function that were significantly associated with carotid stenosis risk. Furthermore, we found a significant gene–gene interaction between *HABP2* rs7923349 and *ITGA2* rs4865756 in affecting the risk of carotid stenosis. This study is expected to identify new targets for the prevention and treatment of carotid artery stenosis and provides a theoretical basis for gene therapy and drug development against new targets in the future.

## Data availability statement

The original contributions presented in the study are publicly available. This data can be found here: https://datadryad.org/, https://doi.org/10.5061/dryad.k98sf7m9r.

## Ethics statement

The studies involving human participants were reviewed and approved by the Ethics Committee of the participating hospitals (The People's Hospital of Deyang City, The Affiliated Hospital of Southwest Medical University, and Suining Central Hospital). The patients/participants provided their written informed consent to participate in this study.

## Author contributions

LL and XY analyzed the data, contributed to method design, and contributed to writing the manuscript. XY, HL, and MY provided data and disease expertise. All authors read and approved the final manuscript.
